# The Potential Role of Auxin and Abscisic Acid Balance and *FtARF2* in the Final Size Determination of Tartary Buckwheat Fruit

**DOI:** 10.3390/ijms19092755

**Published:** 2018-09-13

**Authors:** Moyang Liu, Zhaotang Ma, Tianrun Zheng, Jing Wang, Li Huang, Wenjun Sun, Yanjun Zhang, Weiqiong Jin, Junyi Zhan, Yuntao Cai, Yujia Tang, Qi Wu, Zizhong Tang, Tongliang Bu, Chenglei Li, Hui Chen, Gang Zhao

**Affiliations:** 1 College of Life Science, Sichuan Agricultural University, Ya’an 625014, China; lmyyunxi@163.com (M.L.); WaohUncle_Ma@163.com (Z.M.); kobezey@163.com (T.Z.); wj2366zz@163.com (J.W.); 18428385443@163.com (L.H.); sunnan82475@163.com (W.S.); 15227793373@163.com (Y.Z.); Joan541573@163.com (W.J.); zhanjunyi0412@163.com (J.Z.); caiyt410725@163.com (Y.C.); 15984549383@163.com (Y.T.); wuqi@sicau.edu.cn (Q.W.); 3530279123456789@163.com (Z.T.); tlbu@163.com (T.B.); lichenglei1998@163.com (C.L.); 2 College of Biological Industry, Chengdu University, Chengdu 610101, China

**Keywords:** *Fagopyrum tataricum* Gaertn., abscisic acid, auxin, cell division, embryogenesis, polygonaceae, fruit size, *ARF2*, *SAURs*

## Abstract

Tartary buckwheat is a type of cultivated medicinal and edible crop with good economic and nutritional value. Knowledge of the final fruit size of buckwheat is critical to its yield increase. In this study, the fruit development of two species of Tartary buckwheat in the *Polygonaceae* was analyzed. During fruit development, the size/weight, the contents of auxin (AUX)/abscisic acid (ABA), the number of cells, and the changes of embryo were measured and observed; and the two fruit materials were compared to determine the related mechanisms that affected fruit size and the potential factors that regulated the final fruit size. The early events during embryogenesis greatly influenced the final fruit size, and the difference in fruit growth was primarily due to the difference in the number of cells, implicating the effect of cell division rate. Based on our observations and recent reports, the balance of AUX and ABA might be the key factor that regulated the cell division rate. They induced the response of auxin response factor 2 (*FtARF2*) and downstream small auxin upstream RNA (*FtSAURs*) through hormone signaling pathway to regulate the fruit size of Tartary buckwheat. Further, through the induction of fruit expansion by exogenous auxin, *FtARF2b* was significantly downregulated. The *FtARF2b* is a potential target for molecular breeding or gene editing.

## 1. Introduction

Increasing fruit yield is one of the primary goals of botanists. Fruit size and fruit number are the two complementary agronomic traits that determine the yield of crop varieties. The size of the fruit affects the economic value. From the beginning of crop domestication, the size of the fruit has been the basis for the selection of varieties. For the plant itself, large fruit reserves have more nutrients, which is conducive to the growth and development of crops, faster germination, and resistance to abiotic stress [1].

Possible factors are reported that affect fruit volume [2]. The volume of fruit is the result of the combination of embryo, endosperm, and pericarp [3]. These tissues originate from maternal tissues or different fertilization events (for example, one sperm cell fertilizes diploid central cells to form triploid endosperms, and a second sperm cell fertilizes egg cells to form diploid embryos). These events occur during specific periods of fruit development: cell division occurs primarily at the stage of embryogenesis, whereas cell dilatation occurs more frequently at the stage of filling [4]. In this process, the fertilized egg undergoes a series of cell divisions, leading to different stages of embryonic development (i.e., globular, heart, torpedo, curved cotyledon, and—eventually—mature embryo) [4]. Endogenous hormones play an extremely important role in fruit growth and embryo development. AUX and ABA are the key hormones to control the embryogenesis pattern and promote fruit accumulation in the subsequent filling stage, respectively [5,6,7]. Various genes involved in different mechanisms control fruit size [8]. Because the size of a fruit is generally primarily related to the initial growth of the embryo rather than to the later growth of the embryo [3,9,10], we focused our attention on embryo and cotyledon differentiation, endosperm development, and cell division in the early stages of fruit development [11].

*ARFs* are important transcription factors in the plant auxin signaling pathway, which regulate the expression of auxin-related genes in a positive or negative way, thereby regulating the growth and development of plants [12]. By comparing the protein sequences of the members of tomato, Arabidopsis, potato, rice, and grape *ARF* families, a phylogenetic tree was established, and the *ARF* family was divided into four branches. Among the branches, the protein intermediate region of the subfamily genes of *ARF* I a, II b, III, and IV contains an inhibitory domain, and the *ARF* proteins of these subfamilies are likely transcriptional suppressors [13,14]. *ARF2* is a member of the *ARF* I a subfamily. As a potential auxin negative transcription factor, the gene has been cloned in many types of model plants. In *Arabidopsis thaliana*, *ARF2* mutants exhibit regulatory effects on plant growth and development, including plant enlargement and abnormal tissue morphology [15]. *ARF2* gene is expressed at many stages of plant growth and development. *ARF2* mutants show obvious delayed phenomena in many stages of plant senescence, including flower shedding, leaf senescence, and pod maturation [16]. Some studies find that *ARF2* mutation can promote cell division, and the expression period of *CYCD3.1* and *ant* genes related to the cell cycle in stem and rosette of *ARF2* mutant lines is prolonged accordingly. These results suggest that *ARF2* is an inhibitory factor for cell division and tissue development [17]. *ARF2* also mediates the interaction between auxin and other plant hormones. *ARF2* and *HB33*, as novel regulatory factors, play an important role in regulating plant growth in the AUX and ABA pathways [18].

Tartary buckwheat is the only widely cultivated monomeric total-nutrition grain crop. The essential amino acid composition of the seed protein is not only balanced but also the total content is higher than that of primary grain crops [19,20,21]. With the demand increasing for Tartary buckwheat, understanding the determination of fruit size of Tartary buckwheat has been a key problem. Great differences are found in fruit size among buckwheat varieties, which provide an important genetic resource for understanding how the species regulates fruit size. In this study, we used two Tartary buckwheat varieties with different fruit volumes and similar genetic background and fruit growth cycle to study the factors that lead to different fruit sizes (growth mode, embryo development, cell proliferation) during the development of Tartary buckwheat. Simultaneously, we reported *FtARF2* as a potential target for molecular breeding or gene editing. *FtARF2* was first identified from the Tartary buckwheat genome and then quantified by reverse transcription-quantitative PCR (RT-qPCR) in Tartary buckwheat varieties with different fruit sizes. Finally, by applying exogenous auxin, *FtARF2b* was identified as a potential target for molecular breeding or gene editing.

## 2. Results

### 2.1. Morphological Characteristics of Tartary Buckwheat with Different Types of Fruits and Different Growth Patterns

**Figure 1:**
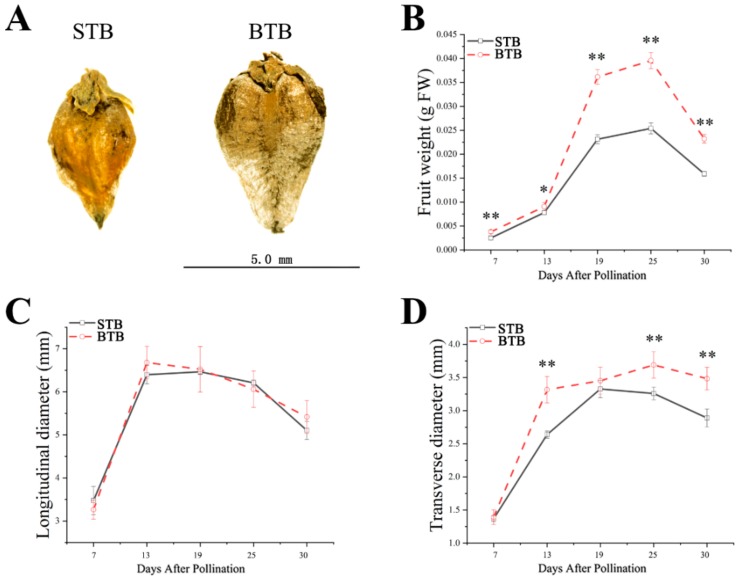
Fruit growth patterns of different accessions of Tartary buckwheat. (**A**) Pictures of mature fruits of the two accessions. (**B**) Average fresh weight of one fruit of the two Tartary buckwheat accessions (STB, black squares; BTB, red circles) is plotted against the developmental stages (days after pollination) (*n* = 5). (**C**) Average longitudinal diameter of one fruit of the two Tartary buckwheat accessions (STB, black squares; BTB, red circles) is plotted against the developmental stages (days after pollination) (*n* = 5). (**D**) Average transverse diameter of one fruit of the two Tartary buckwheat accessions (STB, black squares; BTB, red circles) is plotted against the developmental stages (days after pollination) (*n* = 5); * and ** indicate significant differences (α = 0.05; α = 0.01, least significant difference test (LSD)) among treatments, respectively.

After the different materials were cultivated under the same conditions, we confirmed that the mature fruits of big Tartary buckwheat (BTB) plants were larger than those of small Tartary buckwheat (STB) plants. The mature fruits from BTB were 1.43-fold heavier than those of STB (Figure 1A; Table 1). We also measured the size of mature fruits to determine which two-dimensional axis (i.e., transverse or longitudinal diameter) in the fruits was most affected by the increase in size. The two dimensions both showed an increase in size, with the increase in transverse diameter contributing the most to the increase in fruit size, which resulted in an increase in the size of the final fruit (Table 1). The transverse diameter of the mature fruit of BTB was 1.2-fold longer than that of the STB (Figure 1A; Table 1). Then, the fresh weight and transverse and longitudinal diameters of the two different materials (BTB and STB) were determined from 7 to 30 days after pollination (DAP). We noticed that the growth patterns of the two materials were different. The BTB fruit showed a heavier weight to that of the STB, especially after 13 DAP, increased significantly and remained relatively large throughout the development process (Figure 1B). The growth pattern of the longitudinal diameter of BTB was similar to that of STB (Figure 1C), but the growth pattern of the transverse diameter was different. BTB transverse diameters increased continuously from 7 to 25 DAP and reached the maximum at 25 DAP (Figure 1D). The STB transverse diameters increased from 7 to 19 DAP and reached the maximum at 19 DAP (Figure 1D). Simultaneously, we also found that the growth rate of BTB transverse diameters was higher than that of STB transverse diameters from 7 to 13 DAP (Figure 1D). Therefore, the growth time of the transverse diameters of BTB was not only longer than that of STB but also the growth rate of the transverse diameters of BTB was higher than that of STB transverse diameters at the early stage of fruit development. These growth patterns indicated that the regulatory mechanism(s) conferring the large fruits occurred between 13 and 25 DAP; thus, during the fruit expansion stage.

### 2.2. Anatomical Structure of Tartary Buckwheat with Different Fruit Types

**Figure 2:**
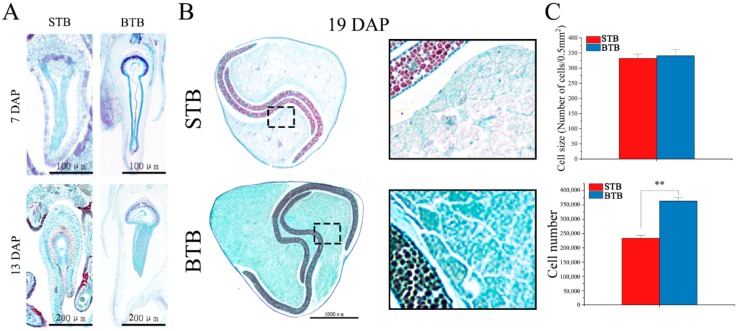
Embryo development of the different accessions. (**A**) Microscopic longitudinal sections of the different accessions of Tartary buckwheat fruits at 7 and 13 DAP. (**B**) Microscopic transverse sections of the different accessions of Tartary buckwheat fruits at 19 DAP (**right panel** is the enlarged view of the boxes in the **left panel**). (**C**) Cell size (0.5 mm^2^) (*n* = 5) and cell number (*n* = 5) in mature fruits of the two accessions; ** indicates significant differences at the level of 0.01.

**Figure 3:**
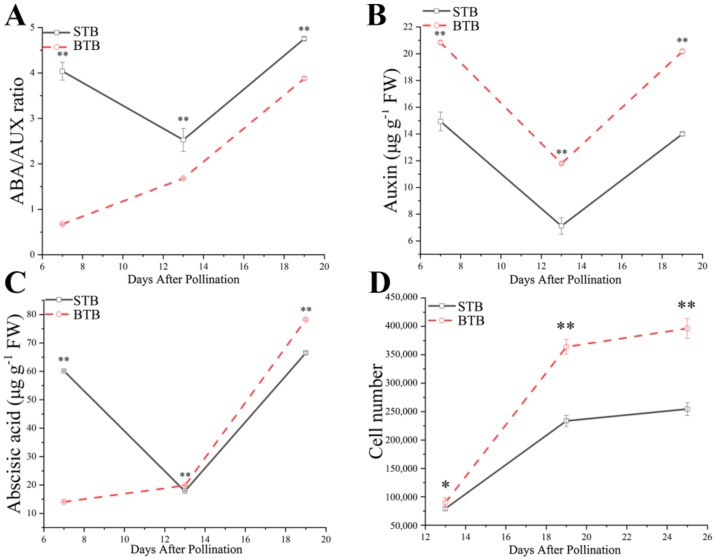
AUX and ABA profiles of the two accessions. (**A**) The corresponding ratios of ABA to AUX from the above figures are plotted against the developmental age of the fruits (DAP) of STB (black squares) and BTB (red circles). (**B**) AUX levels of the two Tartary buckwheat accessions (STB, black squares; BTB, red circles) is plotted against the developmental stages (days after pollination) (*n* = 3); (**C**) ABA levels of the two Tartary buckwheat accessions (STB, black squares; BTB, red circles) is plotted against the developmental stages (days after pollination) (*n* = 3). (**D**) Cell proliferation during 13–25 DAP of fruit development (STB, black squares; BTB, red circles) (*n* = 5); standard deviations are indicated in the different panels; * and ** indicate significant differences (α = 0.05; α = 0.01, LSD) among treatments, respectively.

In the early stage of fruit development, the embryo development of the two different materials was clearly different. The development of STB embryos began earlier than that of BTB embryos. At 7 DAP, the embryo of STB had entered the stage of heart embryo, whereas the embryo of BTB remained in the stage of globular embryo (Figure 2A). At 13 DAP, the embryo of BTB entered the heart stage, and STB preferentially formed the torpedo embryo (Figure 2A). After 19 DAP, both materials showed embryos with full cotyledon development, but the cotyledon of BTB was larger than that of STB by size, and the BTB endosperm was larger than the STB endosperm by size (Figure 2B). Further observation of starch granules in the endosperm showed that starch granules in the BTB endosperm were denser by the density than STB endosperm starch granules (Figure 2B). The BTB fruit was larger than that of STB possibly because of the increase in fruit cell number and/or cell expansion. To identify the primary factors, we measured the embryonic cell number and embryonic cell size of the two materials at 19 DAP. The size of STB cells was similar to that of BTB cells, but the number of BTB cells was significantly greater than that of STB cells (Figure 2C). Based on this result and the determination of the final fruit size at maturation between 13 and 25 DAP, we analyzed the number of embryonic cells in the process of embryogenesis. As shown in Figure 3D, at 13 DAP, the number of cells between BTB and STB was similar, although the embryonic development of BTB remained in the heart stage. Then, from 13 to 19 DAP, the rate of proliferation of BTB cells was significantly higher than that of STB cells. After 19 DAP, the proliferation rate of the two materials was similar, and the increase almost stagnated. Similar to that of fruit at these time points was the pattern of weight gain rate (Figure 1B). According to the above results, we speculate that the development of the early embryo was too rapid, the cotyledon embryo formed prematurely, the cell division cycle was shortened, and the size of the whole embryo was affected. Simultaneously, the early entry into the cotyledon embryo affected the formation of starch granules in the endosperm, thereby affecting the size of the whole fruit.

### 2.3. AUX and ABA Levels Correlate with the Variation in Fruit Size

Endogenous hormones play an extremely important role in fruit growth and embryonic development. AUX and ABA are the key hormones to control the embryogenesis pattern and promote the accumulation of storage products during the subsequent filling stage [5,6], which are closely related to the phenomenon of delayed pre-embryonic development and the many starch grains in the endosperm of BTB. Therefore, the contents of total AUX and ABA during fruit development of the two materials were determined quantitatively. Based on determination of AUX and ABA in the fruits from 7 to 19 DAP by high performance liquid chromatography (HPLC), we observed the accumulative patterns of AUX and ABA in the two materials. The results showed that from 7 to 19 DAP, the accumulative pattern of AUX was the same in the two materials (Figure 3B); whereas the ABA accumulation pattern was different (Figure 3C). The ABA content in the fruit of BTB was relatively low at 7 DAP, and the content in fruit increased with time to 19 DAP (Figure 3C). When STB was at 7 DAP, the ABA content of fruit was relatively high, and the content from 7 to 19 DAP decreased first and then increased (Figure 3C). To summarize, the difference between STB and BTB hormone accumulation models was primarily the difference in ABA accumulation pattern from 7 to 13 DAP. This difference in ABA accumulation patterns led us to compare the ratio of ABA to AUX accumulation from 7 to 13 DAP. We observed that the pattern of BTB and STB was very different, particularly the ratio of ABA to AUX (Figure 3A). The ratio of ABA/AUX in STB was negatively correlated with the fruit development model (Figure 1B) and cell proliferation rate (Figure 3D) from 7 to 13 DAP. From 13 to 19 DAP, the rate of ABA/AUX ratio was similar, and the rate of cell proliferation was similar. Therefore, we hypothesized that at the early stage of fruit development (from 7 to 13 DAP), the high ratio of ABA/AUX accelerated the early entry of embryos into the cotyledon embryo stage, and ABA accelerated embryo maturation (or aging). Under the same ratio of ABA/AUX (from 13 to 19 DAP), the difference in fruit growth power and cell proliferation was presumably due to the response factors of related hormones.

### 2.4. Expression of FtARF2 Genes of Tartary Buckwheat with Different Fruit Types from 13 to 19 DAP

**Figure 4:**
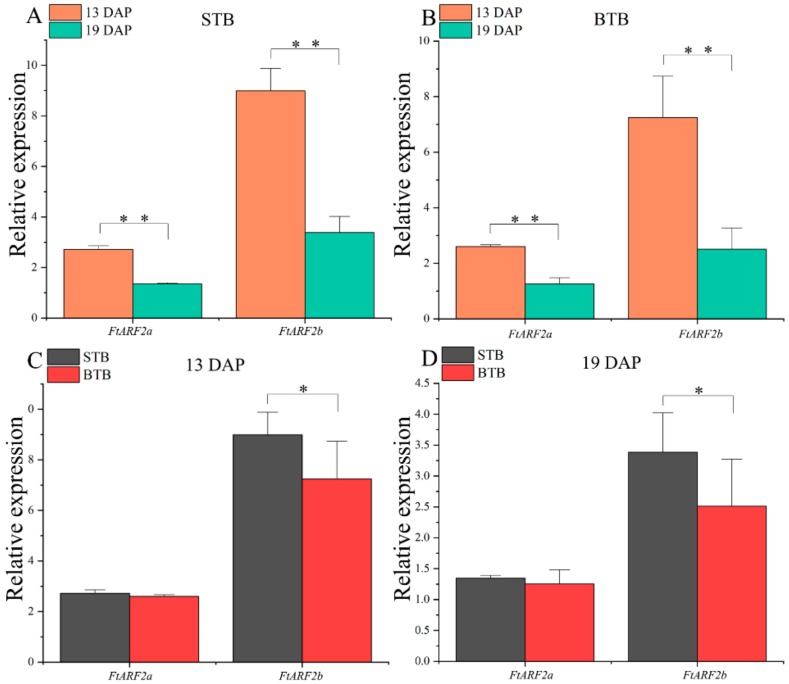
Expression analysis of the two *FtARF2* genes in the different accessions of Tartary buckwheat fruits at 13 and 19 DAP. (**A**) Expression analysis of the two *FtARF2* genes in STB fruit at 13 and 19 DAP. (**B**) Expression analysis of the two *FtARF2* genes in BTB fruit at 13 and 19 DAP. (**C**) Expression analysis of the two *FtARF2* genes in the different accessions of Tartary buckwheat fruits at 13 DAP. (**D**) Expression analysis of the two *FtARF2* genes in the different accessions of Tartary buckwheat fruits at 19 DAP. * and ** indicate significant differences at 0.05 and 0.01 levels, respectively.

Using protein sequences of the Arabidopsis *ARF2* gene as a query, we conducted a BLAST search in the *Fagopyum talaricum* genome browser and obtained *Fagopyum talaricum ARF2* genes (Table 2). Phylogenetic analysis of protein sequences was used to direct the designation of gene names (Figure S1). To investigate the physiological function of *FtARF2* genes, real-time PCR was used to detect the time expression of individual members of the genes. Transcript accumulation was assessed for 2 *FtARF2* genes in the different types of Tartary buckwheat from 13 to 19 DAP (Figure 4). The results showed that the expression patterns of the *FtARF2* genes were similar in the two different types of Tartary buckwheat fruits (Figure 4A,B). The *FtARF2* genes expression decreased from 13 to 19 DAP. The expression of *FtARF2a* in STB and BTB was lower than that of *FtARF2b*, but the decrease in *FtARF2a* was less than that of *FtARF2b* (Figure 4A,B). Therefore, *FtARF2b* had a better negative response than that of *FtARF2a*; and *FtARF2b* had more potential than *FtARF2a* in terms of development (time). We further compared their expression in different types of fruits simultaneously (Figure 4C,D). The results showed that the expression of *FtARF2a* was similar in the different types of fruits in the same period. The expression of *FtARF2b* in STB was higher than that in BTB, particularly at 13 DAP (Figure 4C,D). This finding is consistent with our previous results.

### 2.5. Weight Gain of STB Fruit Induced by AUX

To further understand the response of *FtARF2s* to auxin, different concentrations of AUX (40, 70, 100, 130, or 160 mg L^−1^) were sprayed on the whole plants of STB in the bud stage. The fresh weight of mature fruit of STB increased significantly to 0.0164 g under the action of 100 mg L^−1^ AUX, which was 103% of the blank group (0.0159 g) (Table 3). The application of AUX at concentrations less than or greater than 100 mg L^−1^ had no significant effect on the increase in the weight of the mature fruit of STB and even inhibited the increase in the weight of the fruit. Therefore, 100 mg L^−1^ AUX was the best choice to increase the weight of STB fruit, as we further studied the expression of *FtAFR2* genes in exogenous spraying with AUX.

### 2.6. Expression of the FtARF2 Genes of STB Under AUX Treatment Conditions

**Figure 5:**
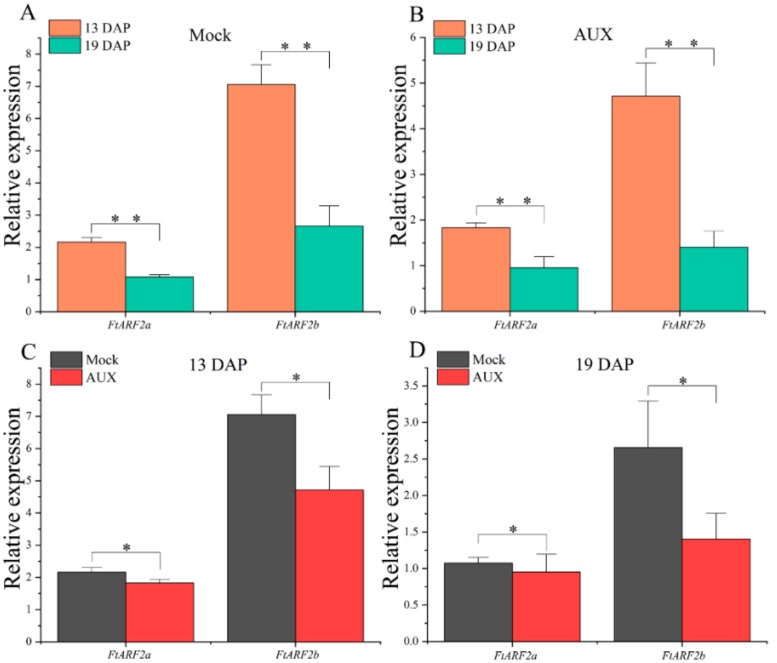
Expression analysis of the two *FtARF2* genes in the STB fruits at 13 and 19 DAP under mock and IAA treatments. (**A**) Expression analysis of the two *FtARF2* genes in the STB fruits at 13 and 19 DAP under mock treatments. (**B**) Expression analysis of the two *FtARF2* genes in the STB fruits at 13 and 19 DAP under IAA treatments. (**C**) Expression analysis of the two *FtARF2* genes in the STB fruits at 13 DAP under mock and IAA treatments. (**D**) Expression analysis of the two *FtARF2* genes in the STB fruits at 19 DAP under mock and IAA treatments. * and ** indicate significant differences at 0.05 and 0.01 levels, respectively.

To further study the response of *FtARF2* genes to auxin in the early stage of development of Tartary buckwheat, STB was treated with 100 mg/L exogenous auxin, to promote the fruit weight gain of Tartary buckwheat. The expression of the *FtARF2* genes was determined by collecting the fruits in the period regulating fruit volume. We found that the expression of the two *FtARF2* genes significantly decreased with fruit development. Compared with the control group, the expression of the *FtARF2b* gene in buckwheat fruit treated with exogenous auxin decreased from 13 to 19 DAP, and *FtARF2a* also showed the same trend, but which decreased more gradually than that in the control group (Figure 5A,B). Simultaneously, compared with the control group, the expression of *FtARF2* genes in buckwheat fruits treated with auxin decreased. The expression of *FtARF2a* and *FtARF2b* decreased significantly at 13 DAP, particularly *FtARF2b*. The expression of *FtARF2a* at 19 DAP was slightly lower than that of the control group, whereas *FtARF2b* showed a significant downward trend (Figure 5C,D). To summarize, *FtARF2* genes were significantly downregulated during 13 to 19 DAP under the action of exogenous auxin. In particular, *FtARF2b* strongly responded to exogenous auxin at 19 DAP. Although the expression of *FtARF2a* was lower under the action of exogenous auxin, *FtARF2b* was better than *FtARF2a* for the sustained negative response of exogenous auxin and the amplitude of the negative response.

### 2.7. FtARF2 Acts Upstream of Fruit Development Genes

**Figure 6:**
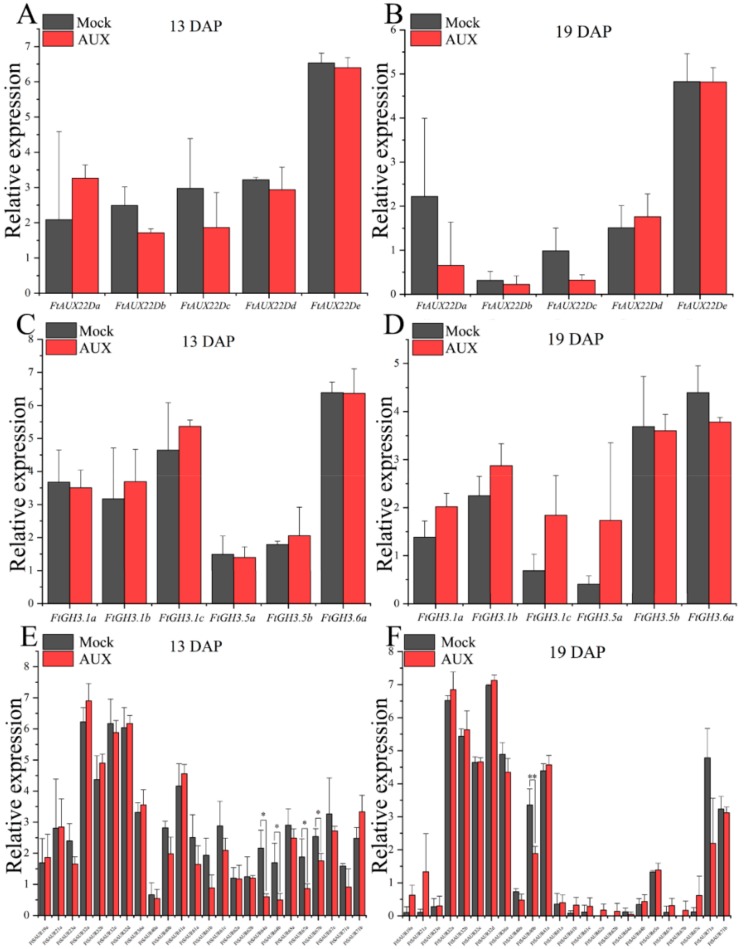
Expression analysis of *FtAux/IAAs*, *FtGH3s*, and *FtSAURs* genes in STB fruits at 13 and 19 DAP under mock and IAA treatments. (**A**) Expression analysis of *FtAux/IAA* genes in STB fruits at 13 DAP under mock and IAA treatments. (**B**) Expression analysis of *FtAux/IAA* genes in STB fruits at 19 DAP under mock and IAA treatments. (**C**) Expression analysis of *FtGH3s* genes in STB fruits at 13 DAP under mock and IAA treatments. (**D**) Expression analysis of *FtGH3s* genes in STB fruits at 19 DAP under mock and IAA treatments. (**E**) Expression analysis of *FtSAURs* genes in STB fruits at 13 DAP under mock and IAA treatments. (**F**) Expression analysis of *FtSAURs* genes in STB fruits at 19 DAP under mock and IAA treatments. * and ** indicate significant differences at 0.05 and 0.01 levels, respectively.

To understand the effect of *FtARF2* on the fruit volume of Tartary buckwheat, we studied the expression patterns of three known downstream genes, auxin/indole acetic acid (*Aux/IAAs*), gretchen hagen 3 (*GH3s*), and *SAURs* [22,23,24], acting on fruit growth in mock and exogenous auxin-treated plants. Using protein sequences of *Arabidopsis Aux/IAAs*, *GH3s*, and *SAURs* genes as a query, we conducted a BLAST search in the *Fagopyum talaricum* genome browser and obtained *Fagopyum talaricum Aux/IAAs*, *GH3s*, and *SAURs* genes, respectively (Figures S2–S4). Real-time PCR was used to detect the expression of individual members of the genes. Transcript accumulation was assessed for those genes in the different types of Tartary buckwheat from 13 to 19 DAP (Figure 6). Compared with the control group, the expression of *FtAux/IAAs* and *FtGH3s* genes in buckwheat fruit treated with exogenous auxin was not significantly different (Figure 6A–D), but significantly downregulated *FtSAURs* genes were detected, including *FtSAUR64a*, *FtSAUR64b*, *FtSAUR67a*, and *FtSAUR67b* at 13 DAP and *FtSAUR40b* at 19 DAP (Figure 6E,F). Thus, auxin decreased the expression of these genes.

## 3. Discussion

### 3.1. Increased Cell Division During Embryogenesis Leads to an Increase in Fruit Size

In this study, based on the analysis of Tartary buckwheat resource information registered in the China Crop Germplasm Resources Information System (http://www.cgris.net/), we selected two Tartary buckwheat varieties as research objects, because their fruits represented large buckwheat and small buckwheat (Table 4). Additionally, their genetic backgrounds were similar, and the fruit development cycle was the same. The size of monocotyledonous fruit is related to the degree of endosperm growth [25,26]. In dicotyledons, the fruit size primarily correlates with the size of the cotyledons, and the number and size of cotyledon cells are directly related to the final fruit size [27]. We observed that Tartary buckwheat had cotyledons and endosperm in mature fruits, which is different from other common crops. Any difference in fruit size has one of two explanations: large fruit contains more cells, indicating a difference in cell division, or larger cells, indicating the expansion of cells (or the combination of these two phenomena). Cell division is active during fruit development and stops before fruit maturation, and cell proliferation primarily occurs during fruit maturation [8]. Additionally, the size of the fruit is generally primarily associated with the initial growth of the endosperm and not with the later growth of the embryo [3,9,10,11]. These results prompted us to compare the changes in cell numbers between STB and BTB in the early stages of fruit development. By comparison, we found that the difference in fruit size was explained by the changes in the number of cells in the fruit (Figure 2), which explained the rate of cell division. Studies on embryogenesis of Tartary buckwheat show that cell division in the embryo reaches a peak from 13 to 19 DAP and then tends to stabilize (Figure 3D). Our observation of STB and BTB embryos is consistent with these results, indicating that the embryonic development of BTB was delayed in the heart phase and torpedo, and that more cells potentially divided than those in STB. The analysis of cell proliferation showed that the cell division time of BTB was longer and the cell division rate was higher after pollination than those of STB, particularly at 13 DAP (Figure 3D). Notably, another example of significant delay in fruit development is described in Arabidopsis *ARF2* mutants [16]. Some studies find that *ARF2* mutation promotes cell division, and the expression period of *CYCD3.1* and ant genes related to the cell cycle in stem and rosette of *ARF2* mutant lines is prolonged accordingly [17]. These results suggest that *ARF2* is an inhibitory factor for cell division and tissue development. For *ARF2* mutants, this delay results in increases in time of embryo growth, cell division activity, and—ultimately—fruit size. A similar mechanism might occur in BTB, which was characterized by delayed early embryogenesis and longer cell proliferation than that of STB.

Although changes in fruit size are the result of cell division and cell expansion, we did not observe changes in cell size area of mature fruit, which indicated that cell division was the primary factor to determine the fruit size of the materials selected in this study.

### 3.2. Regulation of Plant Hormones on Cell Division

Auxin plays an important role in plant growth and development with regulation of the elongation and division of plant cells [28,29]. However, during the development of Tartary buckwheat fruit, a significant positive correlation between auxin and starch was well described [30]. Starch is the primary substance of Tartary buckwheat fruit, which accounts for 70.22% of the total substance content, and is primarily stored in the endosperm of Tartary buckwheat fruit, which has an important effect on the size of fruit [31]. To date, few attempts have been conducted to correlate auxin with cell division in the embryonic development of Tartary buckwheat. *Arabidopsis thaliana ARF2*, a growth hormone responsive gene, is likely a negative regulator of cell division. The mutation of *ARF2* leads to an increase in seed size. This increase suggests that *ARF2* is an inhibitor of cell division and tissue development [17]. In tomato, the overexpression vector of *SlARF2* gene was constructed, and the overexpression of the *SlARF2* gene significantly increased the seed setting rate and reduced the fruit size and weight [32]. These results led us to compare the IAA concentrations of STB and BTB from 7 to 19 DAP and to compare their AUX accumulation patterns; and we found a positive correlation between AUX concentrations and cell division or final seed size. In fact, the concentration of auxin alone might not be sufficient to prevent cell division. Auxin acts as a starting or opening switch and through a secondary regulator. Reducing auxin concentration alone (i.e., removing the on switch) does not reduce other hormones. Based on this assumption, a second ‘off’ switch may be needed to accurately define the time frame for cell division. ABA helps fruits accumulate nutrients during embryonic development, and ABA promotes the absorption of sugar in tomato, strawberry, and citrus [33,34,35] and participates in fruit growth and maturation [36,37]. According to these reports, a small amount of ABA produced from the mother source accumulates before the cell division stops, which may strengthen the concept of ABA as the second closing switch for cell division. Whereas some plants have two ABA peaks during fruit development, others have only one broad peak [38]. Currently, ABA in Tartary buckwheat fruit is negatively correlated with starch and flavonoids contents [30], but nothing else is known. Mechanically, ABA inhibits cell division in two ways. First, ABA induces the expression of cyclin dependent kinase inhibitor (*ICK1*), which leads to block of the cell cycle [39]. For ABA, which also controls the size of the final seed, the second mechanism is to activate the proliferation inhibitor DA1, which is an inhibitory factor of cell proliferation [2]. Based on our research results and related studies, we hypothesized that the high ratio of ABA to IAA at the end of embryonic development (approximately 13 DAP) would result in the division of active cells and be related to the final seed size. This hypothesis was supported by the similarity between the ABA/IAA ratio and fruit volume expansion curve that occurred before 13 DAP (Figure 1B,D and Figure 3A). Compared with BTB, the ABA/IAA ratio of STB was downregulated, whereas that of BTB was upregulated. The growth rate of BTB before 13 DAP was much higher than that of STB. After 13 DAP, although the ABA/IAA ratio pattern tended to be the same, we found that the proliferation patterns of STB and BTB cells were very different. As shown in Figure 3D, the proliferation rate of BTB was much higher than that of STB after 13 DAP. At the end of embryonic development (after 13 DAP), the developmental rate of BTB was different from that of the STB fruit, primarily in terms of weight (accumulation of storage substances). A growth hormone cannot act directly on the target gene and is regulated by binding to the receptor [40]. Additionally, ARF2 also mediates the interaction between auxin and other plant hormones. ARF2 and HB33, as novel regulatory factors, play an important role in regulating plant growth in the auxin and ABA pathways [18]. We speculate that this phenomenon is associated with the signal transduction pathway of related hormones.

### 3.3. ARF2s and Their Downstream SAURs Regulate the Fruit Volume of Tartary Buckwheat at the End of Embryo Maturation

In the current research, the cell signal transduction pathway of plant physiological response to auxin had both a direct mechanism and molecular regulation. A series of genes involved in auxin regulation of transcription include three families of genes (*Aux/IAAs*, *GH3s* and *SAURs*), which contain a conserved sequence “TGTCTC” upstream of their promoters, which are called *AuxREs* [41,42]. *ARFs* are a class of transcription factors that can recognize and bind to *AuxREs* and regulate the expression of auxin responsive genes [41]. In *Arabidopsis thaliana*, *ARF2* mutants exhibit regulatory effects on plant growth and development, including plant enlargement and abnormal tissue morphology [15]. In this study, we identified two *FtARF2* genes from the Tartary buckwheat genome (Figure S1). The expression of *FtARF2* genes was lower in BTB than that in STB and decreased with fruit development in both STB and BTB. The two *FtARF2* genes were negatively correlated with fruit size and development. In *Arabidopsis thaliana*, *ARF2* is an inhibitory factor for cell division and tissue development [17]. Molecular breeding and gene editing require knowledge of specific genes expressed at key stages of growth and development. Because of the existence of many genes in a polygene family, the knowledge of differential expression of individual gene family members has been proven to be crucial [43]. To understand the difference of the two *FtARF2* genes in regulating Tartary buckwheat fruit volume, the volume of STB fruit was increased by exogenous auxin. The expression of *FtARF2* genes was significantly downregulated compared with that of the control group, and negative regulation of the two *FtARF2* genes on the fruit volume of Tartary buckwheat was determined. Based on their positive response, *FtARF2b* was established as a potential target for Tartary buckwheat breeding. Simultaneously, we measured the expression of the downstream genes of *ARFs* (*GH3s*, *AUX/IAAs*, and *SAURs*) between exogenous auxin and blank groups. *FtGH3s* and *FtAUX/IAAs* did not show a significant difference, whereas *FtSAUR64a*, *FtSAUR64b*, *FtSAUR67a*, *FtSAUR67b*, and *FtSAUR40b* in the *FtSAURs* showed significant downregulation. The *SAURs* gene family is unique to plants and the largest family of auxin response factors, which widely exist in various plants [44,45,46,47]. In *Arabidopsis thaliana*, *AtSAUR41* may be related to the size of cells in the resting center and cortex and the transport of auxin and may also be involved in the response to development and environmental signal processes [48]. In rice, *OsSAUR39* inhibits the synthesis and transport of auxin [49]. Notably, although OsSAUR39 and AtSAUR63 are highly homologous, their effects on auxin transport are different. In our results, the significantly expressed *FtSAURs* genes—including *FtSAUR64a*, *FtSAUR64b*, *FtSAUR67a*, *FtSAUR67b*, and *FtSAUR40b*—were downregulated between exogenous auxin and blank groups. However, whether these downstream *FtARFs* target genes were involved in the auxin signaling pathway is uncertain.

### 3.4. Model of Fruit Expansion of Tartary Buckwheat Development: A Time Consideration

**Figure 7:**
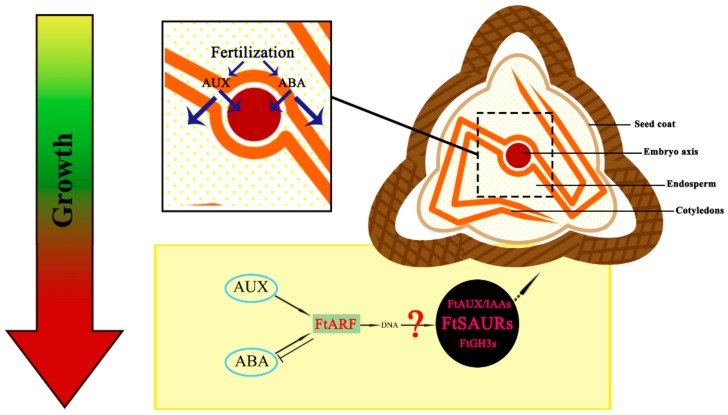
A model of Tartary buckwheat development. The diagram shows the effects of AUX and ABA on embryos shortly after fertilization. Caused by fertilization, the accumulation of AUX and ABA in embryos causes cell division, as shown by the two blue arrows. Induced enlargement is likely to be indirect, through an additional factor, which acts as an inhibitor of expansion. *FtARF2* acts as a negative regulator to reduce the expression of unknown expansion inhibitors. This activates the expression of several “master” regulatory factors and downstream enlargement genes. *FtARF2* apparently receives signals from at least two hormone pathways, including those of AUX and ABA, and its activity is affected by exogenous AUX.

In our model, from fertilization to embryo maturation, the expansion of fruit volume is from the continuous division of embryonic cells, which is strongly influenced by hormones, which may be due to the fertilization potential of plants themselves. The premature maturation of the embryo reduces the cycle of cell division and affects the accumulation of storage materials in later stages, thereby affecting the final volume of the fruit. The balance of AUX and ABA may be the key factors to regulate the cell division cycle. *ARF* has three potential target genes (*AUX/IAA*, *GH*_3_, *SAUR*) in the hormone regulation pathway [22,23,24,50,51]. In Tartary buckwheat, through the response of *ARF2* and downstream *SAURs* in the hormone signaling pathway, the result is the continuous expansion of fruit volume and accumulation of storage materials. However, whether these downstream *FtARFs* target genes (*FtSAURs*) are involved in the auxin signaling pathway is uncertain. To confirm the regulation mechanism of the entire pathway on the fruit volume of Tartary buckwheat, the downstream genes require further study (Figure 7).

Overall, Tartary buckwheat fruit size is undoubtedly dependent on embryonic cell division; nevertheless, significant input is required from several other hormone signaling pathways. The unique role of *FtARF2* in this complex interaction may be the ability to integrate signals, thereby prolonging the cycle of embryonic development, increasing the cycle of cell division and increasing the accumulation of storage materials in fruit tissue.

## 4. Materials and Methods

### 4.1. Plant Material

In 2017, two types of Tartary buckwheat fruits (STB, MIQIAO; BTB, XIQIAO) were collected at the experimental field of the College of Life Science, Sichuan Agricultural University (Lat. 29°97′ N, 102°97′ E, Alt. 580 m), Ya’an, Sichuan, China. Over the past six years, these buckwheat types were introduced into the field and grown in the same ecological environment and cultivation conditions. We observed the development of fruits from anthesis until maturation in April–May 2017. Fruits were collected manually every two days from the beginning of the fruit to maturity, covering a total range of 30 days, and the size and weight of developing fruits were measured. For phytohormone analysis, fruits at five developing stages (7, 13, and 19 DAP) were collected from the same individual, and the fruits of the same developing stage were collected from three replicate plants. The samples were flash frozen in liquid nitrogen and stored at −80 °C for further use.

### 4.2. Light Microscopy

After being dyed by the Safranine and Fast Green double staining method, the samples above were embedded in paraffin and cut in 2 μm sections with a Leica RM2235 paraffin machine (Leica Instruments, Nussloch, Germany). Images of sections were photographed with an Olympus IX83 light microscope (Tokyo, Japan).

### 4.3. Counting Total Number of Cells in Mature Fruits

The fruits were crushed and soaked in water, the fruit coat was removed and the embryo was primarily separated. Four fruits were chopped with a blade and added to a cell wall digestive enzyme mixture of 1 mL (cellulase Onozuka R10 1%, 0.45 M sorbitol, 1 mM KH_2_PO_4_, 10 mM MgCl_2_, 20 mM MES pH 5.6, 0.4% Macerozyme R10 (phytotechnology, Lenexa, KS, USA)). The samples were incubated at 37 °C for 24 h, with a regular smooth vortex. After 24 h of culture, 0.5 mL of cell wall digestive enzyme mixture was added, followed by incubation for 24 h to obtain a uniform dispersed cell mixture. The cells were then diluted to 2 mL and counted on a glass slide in a blood cell counter chamber with 50 µL (Fisher biotech, Pittsburg, PA, USA). According to this value, the total number of cells in 2 mL was calculated, corresponding to the total number of cells in four fruits, which was then divided by 4 to obtain the number of cells in each fruit.

### 4.4. Counting Number of Cells in a Given Area (Cell Size)

Mature fruits were crushed with H_2_SO_4_ for 10 min, washed repeatedly with water, and then soaked for 16 h, put into 20% sucrose solution for 2 h, incubated for 16 h, and embedded in OCT embedding solution (Olympus IX83; Japan). A 15 µm slice was placed in a freeze-microshearing apparatus (Leica, Buffalo Grove, IL, USA), stained in toluidine blue solution, and then washed with water, and the images were collected from the same position and plane at the same magnification. The number of embryonic cells was calculated in a particular area (0.05 mm^−2^).

### 4.5. Phytohormone Analysis

Precisely weighed, approximately 0.5 g of fresh sample was ground in liquid nitrogen. The powders were homogenized in 10 mL of 80% methanol, followed by stirring overnight at 4 °C. Subsequently, this suspension was centrifuged at 12,000 rpm for 10 min under refrigeration (4 °C). The supernatant was collected, and 5 mL of 80% methanol was added to the residue. Similarly, the supernatant was collected after centrifugation. The pooled supernatant (~15 mL) was flash evaporated at 36 °C to no methanol (~3 mL). The distilled bottle was washed with 5 mL of ultrapure water. Then, the rinse was combined with the residual liquid (~3 mL). The solution was decolorized with 15 mL of diethyl ether three times, and the ether phase was abandoned. Aqueous phase was collected and was basified to pH 8.0 with 0.1 M Na_2_HPO_3_. The basified extract was kept on a shaker for 30 min with 50 mg of polyvinylpyrrolidone at 4 °C, which then was centrifuged at 12,000 rpm for 10 min. The supernatant was collected and was acidified to pH 3.0 with 0.2 M citric acid. The solution was partitioned three times against 5 mL of ethylacetate, and the aqueous phase was discarded. The pooled ethylacetate phase (~15 mL) was flash evaporated at 36 °C to near-dryness. The residue was dissolved in 1 mL of methanol [52,53,54].

The sample was filtered through a nylon 66 filter (25 mm diameter, 0.45 μm pore size) before injection for high-performance liquid chromatography (HPLC). HPLC analysis was performed on an Agilent 1260 system using a C18-ODS (3.5 µm × 150 mm × 4.6 mm) column (Agilent, Santa Clara, CA USA) and a UV/VIS detector. Injection volume of 10 µL, column temperature of 35 °C, flow rate of 1 mL min^−1^, and run time of 10 min were maintained for all analyses. The system was calibrated with external standards of AUX and ABA. For detecting these compounds, the separation was performed with a mixture of methanol and distilled water containing 0.6% acetic acid (*V*:*V* = 50:50) with the following isocratic elution. The eluent was scanned at 257 nm.

### 4.6. AUX Treatment of STB

In the bud stage, Tartary buckwheat with a similar growth state were sprayed once with 40, 70, 100, 130, or 160 mg L^−1^ of indoleacetic acid (IAA), and the same amount of water was sprayed as the control (Mock). When fully ripe, the weight of the fruits was measured. At 13 and 19 DAP, the fruit samples were rapidly frozen in liquid nitrogen and stored at −80 °C for further use.

### 4.7. Gene Identification and Phylogenetic Analysis

The Tartary buckwheat genome was downloaded from the Tartary Buckwheat Genome Project (TBGP; http://www.mbkbase.org/Pinku1/). The *ARF2* gene of Tartary buckwheat were identified by BLASTP search (https://blast.ncbi.nlm.nih.gov/Blast.cgi). All known Arabidopsis *ARF2* gene was used to query the initial protein on TBGP website, and the candidate genes were identified by BLASTP search at a score value of ≥100 and *e* -value ≤ 1 × 10^−10^ [55]. The sequence length, molecular weight, isoelectric point, and subcellular localization of the identified *FtARF2* protein were obtained by using the tools of ExPasy website (http://web.expasy.org/protparam/). The phylogenetic trees were inferred using neighbor-joining (NJ) method of Geneious R11, with the following parameters: Jukes–Cantor model, global alignment with free end gaps, and Blosum62 cost matrix. *ARF2* protein sequences from *Arabidopsis thaliana* were downloaded from UniProt database (https:/www.uniprot.org). *FtAux/IAAs*, *FtGH3s* and *FtSAURs* were obtained in the same procedure.

### 4.8. Real-Time PCR Confirmation of Differentially Expressed Genes

Quantitative real-time PCR analysis was performed to confirm the expression pattern of each identified gene. The corresponding sequences of these genes were obtained from the Tartary buckwheat (Pinku1) genome sequence database (http://www.mbkbase.org/Pinku1/). The RT-qPCR primers were designed according to the transcript sequences of these genes using Primer3 software (http://frodo.wi.mit.edu/) (Table 5). The *FtH3* gene was used as the internal control. RT-qPCR experiments were replicated at least three times.

First-strand cDNA was synthesized from 1 mg of DNase I-treated RNA samples in a 40 μL reaction solution with random primers, using a PrimeScript RT Reagent Kit with gDNA Eraser (TaKaRa, Tokyo, Japan). Standard RT–qPCR was performed using SYBR Premix Ex Taq II (TaKaRa, Tokyo, Japan) on a CFX96 Real Time System (BioRad, Hercules, CA, USA). Data were analyzed by the 2^−(∆∆*C*t)^ method to obtain relative mRNA expression data [56].

### 4.9. Statistical Analysis

All the data were analyzed by analysis of variance using the Origin Pro 2018b (OriginLab Corporation., Northampton, MA, USA) statistics program, and the means were compared by the least significant difference test (LSD) at the 0.05 and 0.01 level of significance.

## Figures and Tables

**Table 1:** Plant materials used in this study and their characteristic mature fruit size and weight

Materials	Transverse Diameter (mm)	Longitudinal Diameter (mm)	Weight (g)
BTB	3.48 (±0.17)	5.42 (±0.38)	0.023 (±0.00 087)
STB	2.89 (±0.14)	5.10 (±0.21)	0.016 (±0.00 051)
BTB/STB ratio	120%	106%	143%

**Table 2:** *FtARF2* genes encoding ARF proteins along with their molecular details

Gene	Gene ID	Protein Length	Mw (kDa)	pI	Location	Domain	Homologous	E-Value	Similarity	Localization
*FtARF2.01*	FtPinG0002469700.01	766	81	6.03	Ft1	DBD, ARF, CTD	*AtARF2*	8 × 10^−50^	79	Nucleus
*FtARF2.02*	FtPinG0005575800.01	721	85	6.03	Ft2	DBD, ARF, CTD	*AtARF2*	1 × 10^−42^	79	Nucleus

**Table 3:** Effects of IAA on the fruit of STB

Materials	IAA Concentration (mg L^−1^)	Weight (g)
STB	0 (Mock)	0.015 90 (±0.00 051) ad
	40	0.015 94 (±0.00 041) ab
	70	0.014 82 (±0.00 075) cd
	100	0.016 44 (±0.00 055) a
	130	0.015 38 (±0.00 059) bc
	160	0.014 56 (±0.00 060) d

Small letter(s) indicate significant differences (α = 0.05, LSD) among treatments.

**Table 4:** Mature fruit size, shape, and weight of the different accessions from *Fagopyrum tataricum*.

No.	Cultivar	Size	Shape	Weight (g)
1	87-23 (F6080)	Big	Peach shape	0.0257
2	HEI LI MI QIAO	Big	Long shape	0.0244
3	87-26 (F6245)	Big	Long cone	0.0232
4	LIAO QIAO75 HUAO (KU)	Big	Long shape	0.0231
**5**	**XI QIAO**	**Big**	**Long shape**	**0.0230**
6	87-27 (F6273)	Big	Long cone	0.0230
7	HEI ZI QIAO	Medium	Wide	0.0228
8	Mai QIAO	Medium	Long shape	0.0218
9	87-14 (F3091)	Medium	Long cone	0.0218
10	YUAN ZI QIAO	Medium	Long shape	0.0214
11	BIAN ZI QIAO	Medium	Long shape	0.0207
12	YANG QU KU QIAO	Medium	Long shape	0.0203
13	KU QIAO (TUO YUAN)	Medium	Long shape	0.0203
14	XI YANG KU QIAO	Medium	Long shape	0.0199
15	SHI CHE E	Medium	Long shape	0.0196
16	JIU JIANG KU QIAO	Medium	Long shape	0.0195
17	PING DING KU QIAO	Medium	Long shape	0.0194
18	MA KU QIAO	Medium	Wide	0.0193
19	E LUO WU QIE	Medium	Long shape	0.0192
20	KU TIAO ZI	Medium	Long shape	0.0190
21	KU BING QIAO	Medium	Wide	0.0187
22	KU QIAO MAI	Small	Long shape	0.0160
**23**	**MI QIAO**	**Small**	**Long shape**	**0.0160**
24	ER BAI KU QIAO	Small	Long shape	0.0158
25	88-33 (IV-171)	Small	Short cone	0.0154
26	LENG FAN TUAN	Small	Long shape	0.0151
27	HEI KU QIAO	Small	Wide	0.0150
28	HONG XI KU QIAO	Small	Long shape	0.0150
29	MA QIAO	Small	Long cone	0.0149
30	HUI CHA KU QIAO	Small	Wide	0.0148
31	82-4-6	Small	Long shape	0.0146
32	XI KU QIAO	Small	Long shape	0.0140
33	82-8-1	Small	Long shape	0.0130
34	LV QIAO	Small	Long shape	0.0100

Bold samples are the test materials.

**Table 5:** Primers of sequences.

Gene ID	Forward Primer (5′–3′)	Reverse Primer (5′–3′)
*FtARF2.01*	ACCTTCCGTTTCTCCACTGACA	GACCCTTGATAATGATAACCCACTT
*FtARF2.02*	AGACTTGTGGCTGGTGACGCT	GCTAGATATGACTGACGAGGGAACT
*FtGH3.1a*	AAGTTGGTGGACATGGTTGACG	TGCGGAGCAGAGTTGTGGAA
*FtGH3.1b*	TTCCTCCACCTTCATTACCCG	GATACCTTCCTCCCAGTTCTCCTT
*FtGH3.1c*	CGGAACTCGCTGACTTTATCATATC	AGGGAGACCACCGCTGTAGAA
*FtGH3.5a*	CGTTGTGCTTCCTCAAATCGG	TCGCCAACCTCAACCTCAGTC
*FtGH3.5b*	CGGACTCGTACCAAAGCATGT	CTTGATAGCACGAATGAACCCA
*FtGH3.6a*	AGATGCGTCAAGTTCAAGCCA	TGCTTATGACCAGGAACCCACT
*FtSAUR19a*	TCAAAGCCATACTCCTCTTCAGC	TATGGTGGGGCTCGAAGGA
*FtSAUR21a*	GCACTTTGCGGTGTATGTAGGC	TTGTTCATCACAGGGAATGGTTAG
*FtSAUR23a*	GGGTGGATGGGTATTTGGGTG	GCCGCCTTGAGGAGGTTGAT
*FtSAUR32a*	CGATTACGATCCCTTGCCATG	TCAAGCCCTAAAACACCCAACA
*FtSAUR32b*	GAGAGGAGATGCGGCGATTC	CGAAGTACGCCTTTCTGCTGG
*FtSAUR32c*	ATGGCGATTATGCGAAAGCT	ACGATCACCGGAACATACCCT
*FtSAUR32d*	GGTTTCTTATTCCGACTCAGTTCAT	GAATCTCCCTTAAACAATCTGGCT
*FtSAUR36a*	GTCATTCCGAGGTCACCAACAA	AACCCACGGCGGCTTTATG
*FtSAUR40a*	GGACACGAAGCAGAGGAGCA	TTGAGCGACCAAGAACTGAACG
*FtSAUR40b*	ATCTGGCGGTTGATGTGGG	GATACGGGGCAAGGGATAGTG
*FtSAUR41a*	ATTGGACATCCGTCTATTATTGCTC	TAATCTGCCTGAAAGAGTCCACG
*FtSAUR61a*	TTGCCTTCTGTTATCTCAACCACC	CATACCTTTGCTTATCCTCATCGTC
*FtSAUR61b*	TTCCGTTTGCCCTCTGTGAT	GTAAGACAGCGGCACCACATAA
*FtSAUR61c*	TGAGAAGGGCCAGTTTGTCGT	ACTTGCCAGCCCAAACTCTTC
*FtSAUR62a*	TTACACTTCCCTTGTCTTATCTCGG	GGATCAATGCTTGCTCTAGTTCTCT
*FtSAUR62b*	GAGAAGTAACAACACCAAGCAATCA	ATAAGACAAGGGAAGTGTAAAGCGT
*FtSAUR64a*	GAAGAGTTTGGGTTGGTTGGTG	GACACAATCAACGCTTCTTCCAA
*FtSAUR64b*	ATGGCAGAAGAGGAGTTTGGATT	GAGTTCTCTAGCTGCGTTCCGAT
*FtSAUR65a*	ACTTTGTGGTCTACACCGCTGAT	AACTCCTCCTCCGCCATTCTA
*FtSAUR67a*	GCAGTGGCAAGTAGGAAGAGGA	GAGATTTGAGATACACCAAGGGAAG
*FtSAUR67b*	CCAAGGAGCAACAGCACCAA	CAAACTCCTCTTCCGCCATTCT
*FtSAUR67c*	TTGGCAAGAAAGTGGAGAAAGC	CCGTGTAGACCATAAACTGACCCT
*FtSAUR71a*	CATTTCCAAGAAGCAACAACACC	TCCAAACTCCTCTTCTGCCATT
*FtSAUR71b*	CAACAGCACCAACAAATCATCAAC	CAAACTCCTCTTCGGCCATTC
*FtAUX22Da*	GTGTTGGAAAGCATGTTTAAGGTG	CGAAGCCTCTTACACGACGACA
*FtAUX22Db*	AGTGAGCTACTAGAGGCATTGGAAG	CCACGGAACATCACCAACAAG
*FtAUX22Dc*	CAAGGCTTACAAGAGCTACATCGA	AACATTTCCCACGGCACATC
*FtAUX22Dd*	GGATCGACCGACGACCACTT	GCCATCCAACTATTTGTGCCTT
*FtAUX22De*	AGATCAACACTCAACCCTCTGCTT	GCTCCGTCCATGCTTACTTTCAC
*FtH3*	GAAATTCGCAAGTACCAGAAGAG	CCAACAAGGTATGCCTCAGC

## Supplementary Materials

Supplementary materials can be found at http://www.mdpi.com/1422-0067/19/9/2755/s1.

## Abbreviations

ABA	abscisic acid
ARF2	auxin response factor 2
AUX	auxin
Aux/IAAs	auxin/indole acetic acid
BTB	big Tartary buckwheat
DAP	days after pollination
HPLC	high performance liquid chromatography
GH3s	Gretchen Hagen 3
IAA	indoleacetic acid
ICK1	cyclin dependent kinase inhibitor
RT-qPCR	reverse transcription-quantitative PCR
SAURs	small auxin up RNAs
STB	small Tartary buckwheat
