# Supplementary figures and images for: The Potential Role of Auxin and Abscisic Acid Balance and FtARF2 in the Final Size Determination of Tartary Buckwheat Fruit
Source: Int J Mol Sci. 2018 Sep 13;19(9):2755. doi: 10.3390/ijms19092755 (PMC6163771; doi:10.3390/ijms19092755)

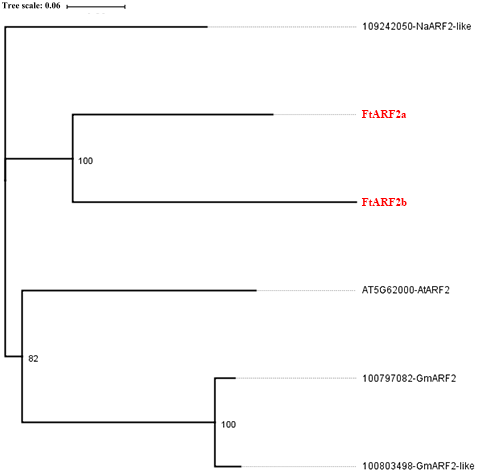

Supplement: Supplementary file 1 [file ijms-19-02755-s001.zip › ijms-340055-supplementary/Supplemental Figure 1.tif]

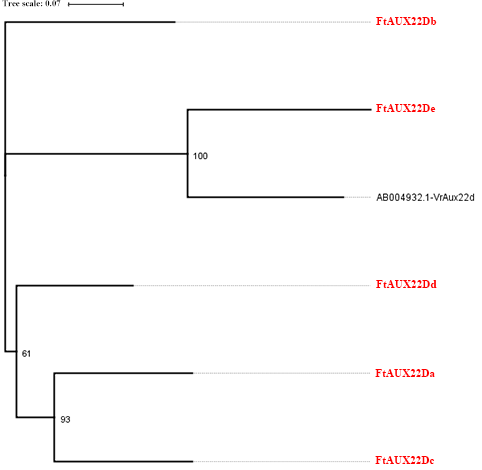

Supplement: Supplementary file 1 [file ijms-19-02755-s001.zip › ijms-340055-supplementary/Supplemental Figure 2.tif]

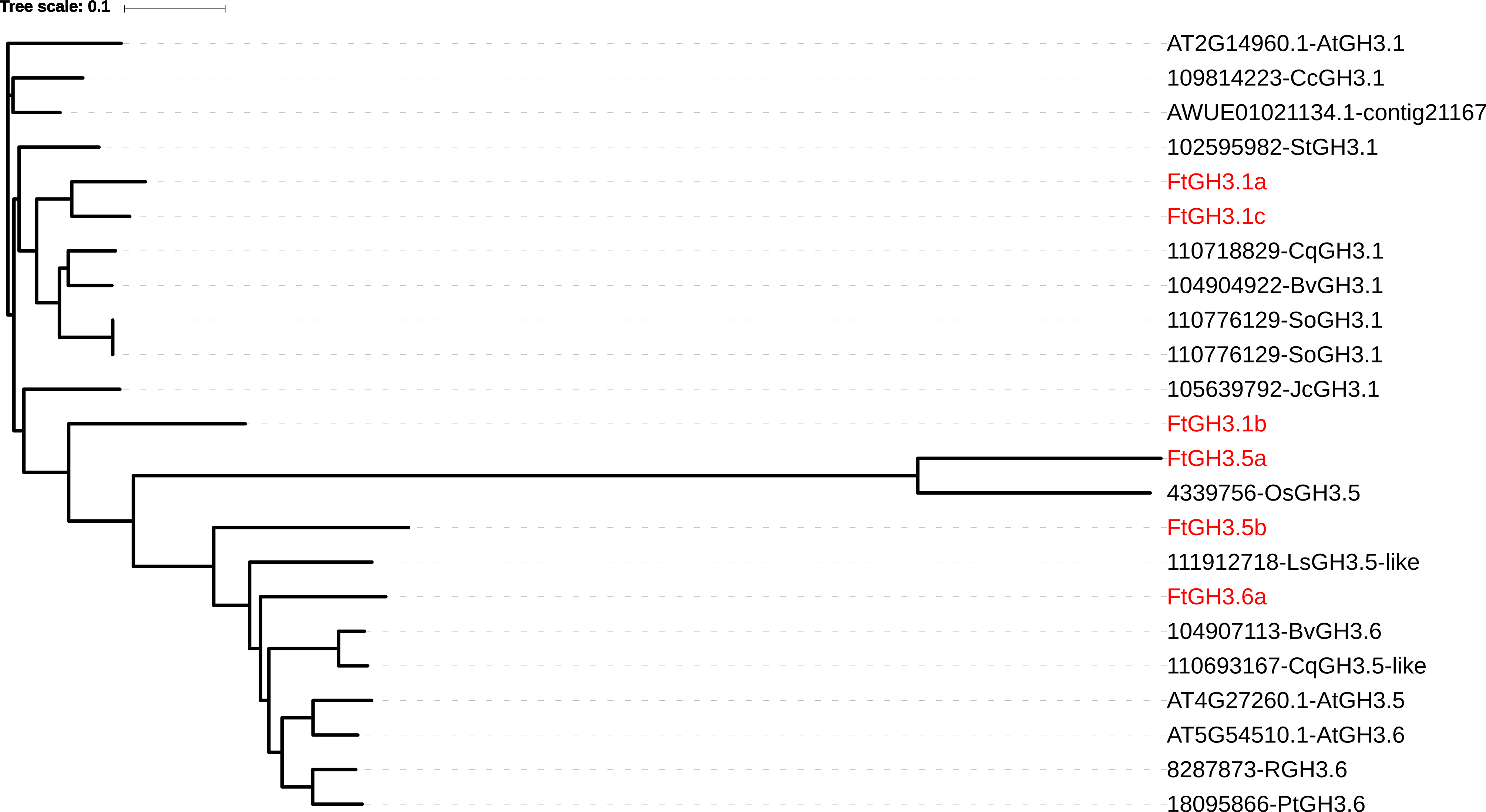

Supplement: Supplementary file 1 [file ijms-19-02755-s001.zip › ijms-340055-supplementary/Supplemental Figure 3.tif]

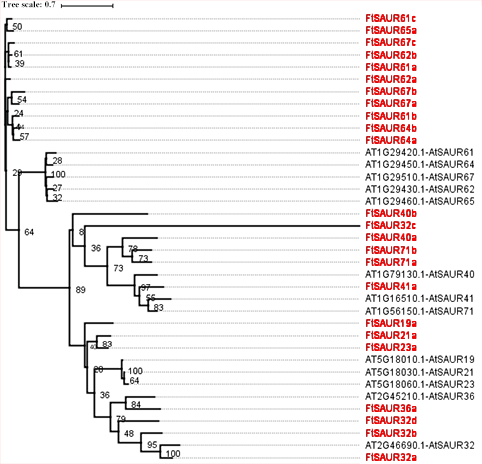

Supplement: Supplementary file 1 [file ijms-19-02755-s001.zip › ijms-340055-supplementary/Supplemental Figure 4.tif]
